# Oral S-ketamine effective after deep brain stimulation in severe treatment-resistant depression and extensive comorbidities

**DOI:** 10.1136/bcr-2020-238135

**Published:** 2021-01-25

**Authors:** Jolien K E Veraart, Jeanine Kamphuis, Mathis Schlegel, Robert A Schoevers

**Affiliations:** 1 Department of Psychiatry, University of Groningen, University Medical Centre Groningen, Groningen, The Netherlands; 2 PsyQ Haaglanden, Parnassia Psychiatric Institute, The Hague, The Netherlands; 3 Research School of Behavioural and Cognitive Neurosciences (BCN), University of Groningen, Groningen, The Netherlands

**Keywords:** therapeutic indications, depressive disorder

## Abstract

This case report describes successful maintenance treatment with oral S-ketamine in a patient with severe depression, who previously was resistant to electroconvulsive therapy and deep brain stimulation, and who also had comorbid psychotic and obsessive compulsive symptoms.

## Background

Treatment resistance in patients with depression is associated with vast losses in quality of life and an increased risk of suicidal ideation and suicide.^1^ Infusions of the N-methyl-D-aspartate receptor antagonist ketamine or the enantiomer S-ketamine have shown rapid antidepressant effects in treatment-resistant depression (TRD).^2^ Recent studies suggest efficacy plus good tolerability of repeated oral ketamine treatment.^3 4^ Patients with comorbid psychiatric disorders are often excluded from clinical trials, although they may represent the most severe TRD cases. The case presented here suggests that remission of severe TRD with extensive comorbidities can be achieved with repeated administration of oral S-ketamine when electroconvulsive therapy (ECT) and deep brain stimulation (DBS) have been ineffective.

## Case presentation

Our patient is a 55-year-old female who presented to our academic centre with severe TRD, comorbid obsessive-compulsive disorder (OCD) and psychotic symptoms (auditory hallucinations). Subsequent treatment strategies, including extensive pharmacotherapy (serotonin–norepinephrine reuptake inhibitors, tricyclic antidepressants and lithium addition), psychotherapies (cognitive behavioural and systemic therapies) and ECT had not significantly improved her situation for over 20 years.

In 2014, she participated in a clinical trial of DBS. She received bilateral implants of four contact electrodes targeting the ventral anterior limb of the internal capsule.^5^ Various adjustments of electrode settings failed to improve her situation, leading to despair and increase in suicidal ideation.

## Treatment

Augmentation with generic oral S-ketamine from our hospital pharmacy was initiated after the patient gave written informed consent for off-label ‘compassionate use’ treatment. Her other medications remained unchanged: venlafaxine 300 mg, clozapine 450 mg, glycopyrronium 0.7 mg and movicolon daily, plus 5 mg nitrazepam three times per week. DBS settings were kept stable at 3.0 V, pulse width 60 and frequency 180 Hz.

Oral S-ketamine treatment started at 0.5 mg/kg twice weekly and was titrated to 2.0 mg/kg. Vital signs were stable. Apart from temporary dizziness, no adverse events occurred.

## Outcome and follow-up

**Figure 1:**
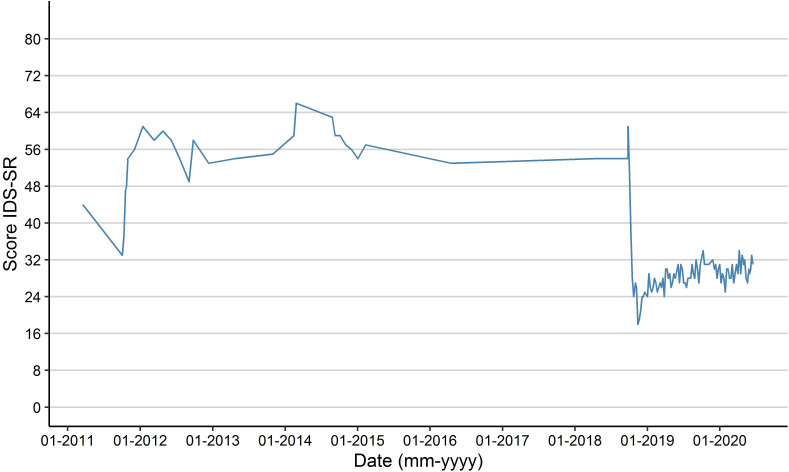
IDS-SR (Inventory of Depressive Symptomatology Self-Report) scores.

Evaluation after 6 weeks revealed a decrease in Inventory of Depressive Symptomatology Self-Report (IDS-SR) score from 54 to 30 and Hamilton Depression Rating Scale 17 score from 24 to 6 (figure 1 for IDS-SR scores). The patient reported an overall good response and started to function again in important domains of life. Interestingly, both her auditory hallucinations and obsessive-compulsive symptoms decreased. She currently continues S-ketamine treatment twice weekly at home, and has been in remission for 18 months now. Risks and benefits of prolonged treatment were extensively discussed with the patient. We systematically monitor symptoms and possible adverse effects.

## Discussion

We believe this case illustrates the potential of generic oral S-ketamine in the most severe cases of TRD. The oral S-ketamine showed beneficial effects not only on the depressive symptoms, but also on the disturbing comorbid obsessive-compulsive and psychotic symptoms in this case. Significant improvement in obsessions after ketamine administration in patients with OCD has been reported previously in a small randomised controlled crossover trial.^6^ Patients with psychotic symptoms have been excluded for most clinical trials studying ketamine as a treatment for depression. Ketamine has the potential to induce psychotomimetic effects in healthy subjects and to exacerbate psychotic symptoms in patients with schizophrenia.^7^ However, the psychotic manifestations, such as perceptual abnormalities, disordered thought and dissociation after ketamine administration are transient and seem to be well tolerated, even in patients with psychotic vulnerability.^8–10^ Of note, ketamine is also suggested to be more effective in patients with a higher childhood trauma burden,^11^ which is a known risk factor for the development of TRD and suicide.^12^

Repeated oral S-ketamine was safely combined with other psychiatric drugs and even with the experimental neuromodulation treatment of DBS. Safety and tolerability of oral S-ketamine as add-on to standard antidepressants has been previously described in a case series by Paslakis *et al*.^13^ S-ketamine augmentation via other routes of administration has also been studied in combination with regular antidepressants and antipsychotics.^13–15^ However, to the best of our knowledge, this is the first paper reporting on S-ketamine administration in a patient with DBS implants.

Given the enormous burden of TRD for patients, we believe trials with (S-)ketamine treatment in patients with extensive comorbidities are both needed and justified, if outcomes are thoroughly monitored over time. Careful monitoring of patients eligible for last resort ‘compassionate use’ programmes will provide useful information on the treatment potential of (S-)ketamine to relieve suffering and improve quality of life.

**Patient’s perspective**

Since the ketamine treatment, my mood changed. I am able to enjoy activities again. I started feeling emotions again that I have not felt since my depression started, such as enjoyment, sadness and missing my dog. My obsessive and compulsive symptoms have decreased since this mood improvement; they are still there but I can cope now.

**Learning points**

- Maintenance treatment with oral S-ketamine showed long-term beneficial effects in severe treatment-resistant depression (TRD).
- Oral S-ketamine reduced comorbid psychotic symptoms and obsessive-compulsive symptoms in a patient with TRD.
- Oral S-ketamine treatment was safely combined with experimental deep brain stimulation for depression.
